# Accuracy of taxonomy prediction for 16S rRNA and fungal ITS sequences

**DOI:** 10.7717/peerj.4652

**Published:** 2018-04-18

**Authors:** Robert C. Edgar

**Affiliations:** Sonoma, CA, USA

**Keywords:** Microbiome, Taxonomy, Algorithm, Benchmark

## Abstract

Prediction of taxonomy for marker gene sequences such as 16S ribosomal RNA (rRNA) is a fundamental task in microbiology. Most experimentally observed sequences are diverged from reference sequences of authoritatively named organisms, creating a challenge for prediction methods. I assessed the accuracy of several algorithms using cross-validation by identity, a new benchmark strategy which explicitly models the variation in distances between query sequences and the closest entry in a reference database. When the accuracy of genus predictions was averaged over a representative range of identities with the reference database (100%, 99%, 97%, 95% and 90%), all tested methods had ≤50% accuracy on the currently-popular V4 region of 16S rRNA. Accuracy was found to fall rapidly with identity; for example, better methods were found to have V4 genus prediction accuracy of ∼100% at 100% identity but ∼50% at 97% identity. The relationship between identity and taxonomy was quantified as the probability that a rank is the lowest shared by a pair of sequences with a given pair-wise identity. With the V4 region, 95% identity was found to be a twilight zone where taxonomy is highly ambiguous because the probabilities that the lowest shared rank between pairs of sequences is genus, family, order or class are approximately equal.

## Background

Next-generation sequencing of tags such as the 16S ribosomal RNA (rRNA) gene and fungal internal transcribed spacer (ITS) region has revolutionized the study of microbial communities in environments ranging from the human body (Cho & Blaser, 2012; Pflughoeft & Versalovic, 2012) to oceans (Moran, 2015) and soils (Hartmann et al., 2014). A fundamental step in such studies is to predict the taxonomy of sequences found in the reads. Many taxonomy prediction algorithms have been developed, including the RDP Naive Bayesian Classifier (NBC) (Wang et al., 2007), GAST (Huse et al., 2008), the lowest common ancestor (LCA) method in MEGAN (Mitra, Stärk & Huson, 2011), 16Sclassifier (Chaudhary et al., 2015), SPINGO (Allard et al., 2015), Metaxa2 (Bengtsson-Palme et al., 2015), SINTAX (Edgar, 2016), PROTAX (Somervuo et al., 2016), microclass (Liland, Vinje & Snipen, 2017), and methods implemented by the mothur (Schloss et al., 2009), QIIME v1 (Caporaso et al., 2010) and QIIME v2 (https://qiime2.org) packages. A few studies have utilized tree placement algorithms such as pplacer (Matsen, Kodner & Armbrust, 2010) and RaxML (Stamatakis, 2014). Specialized sequence databases providing taxonomy annotations include UNITE (Nilsson et al., 2015) for fungal ITS and RDP (Maidak et al., 2001), Greengenes (DeSantis et al., 2006) and SILVA (Pruesse et al., 2007) for 16S rRNA. Most taxonomies in the RDP database were predicted by the RDP NBC, while most taxonomies in Greengenes and SILVA were annotated by a combination of database-specific computational prediction methods and manual curation (McDonald et al., 2012; Yilmaz et al., 2014).

### Previous taxonomy benchmarks

Previous approaches to validating taxonomy prediction methods are briefly summarized below; this is not intended to be a comprehensive survey.

#### Leave-none-out

The leave-none-out (LNO) strategy uses the complete reference database as both the test set and training set. Test sequences may be modified by extracting a short segment or by adding simulated noise. In Werner et al. (2012), LNO was used to evaluate coverage, defined as the fraction of sequences that are classified, without attempting to assess whether predictions are correct. This approach is flawed because a method could have higher coverage due to a higher false positive rate.

#### Leave-one-out

With leave-one-out (L1O), each sequence in the reference database is extracted for use as a query which is classified using the remaining reference sequences as a training set (Wang et al., 2007; Deshpande et al., 2015; Edgar, 2016). In these papers, accuracy is defined as the fraction of sequences that are correctly classified at each rank (see *Acc*_RDP_ in Methods).

#### Leave-clade-out

With leave-clade-out (LCO) cross-validation (Brady & Salzberg, 2009), test and training sets are constructed so that every taxon at a given rank (the *excluded* rank) is present in either the test set or training set, but not both. This models a scenario where all query sequences belong to unknown taxa at the excluded rank and below. In Edgar (2014), this approach was used to measure accuracy of 16S rRNA and ITS taxonomy predictions using novel, taxonomy-specific metrics including *under-classification* and *over-classification* (see Methods for definitions). The accuracy of several methods was measured for all possible pairs of excluded rank and predicted rank; e.g., family prediction accuracy when genus is excluded. In Deshpande et al. (2015), the lowest rank was excluded (species or genus, depending on the training set) and predictions were evaluated only at the parent rank (genus and family, respectively). The “Novel taxa evaluation” of Bokulich et al. (2017) uses a similar approach to Edgar (2014). In Edgar (2016), test/training pairs were obtained by excluding some families and some genera to model a more realistic scenario where some low ranks are novel and most high ranks are known.

#### K-fold cross-validation

In Lan et al. (2012), two- and five-fold cross-validation was used in which the reference database was randomly divided into test and training sets of relative size 1/2 (two-fold) or 4/5 and 1/5 (five-fold). With a training set containing only 1/5 of the reference sequences, many sequences in the test set belong to taxa that are missing from that training set, enabling evaluation of performance on novel query sequences. Ten-fold cross-validation was used by Allard et al. (2015) and Bokulich et al. (2017) where 10% of the reference sequences were extracted to use as a test set; in the latter work the test/training pairs were stratified such that 10% of the sequences for each taxon was assigned to the test set.

#### Mock community validation

Reference sequences and reads from artificial (*mock*) communities containing known strains have also been used for taxonomy validation (Allard et al., 2015; Edgar, 2017a; Bokulich et al., 2017).

#### BLAST top hit

In Chaudhary et al. (2015), the taxonomy of the top BLAST hit is used as a truth standard for assessment of predictions by 16Sclassifier. This approach is flawed because the top BLAST hit does not necessarily have the same taxonomy as the query sequence.

### Unrealistic benchmark tests

Ideally, benchmarks would be designed to model prediction tasks encountered in practice. However, with L1O, LNO and *k*-fold cross-validation, test sequences generally have high similarity to the training sets, and most taxa are therefore known. This scenario is unrealistic because in practice, query sequences often have low similarities to reference sequences (Caporaso et al., 2011; Hibbett et al., 2016), and many of them therefore do not belong to a named genus, reflecting that only ∼2% of prokaryotic genera have so far been classified by taxonomists (Yarza et al., 2014). With LCO, novel taxa are present in a test set, and lower identities arise as a side effect of excluding groups. This approach is unrealistic because in practice it is not known whether or not a given rank of an environmental sequence is novel. Mock communities are unrealistic models of typical environmental samples because they are composed of type strains, and all sequences are therefore both known and named.

### Cross-validation by identity

**Figure 1:**
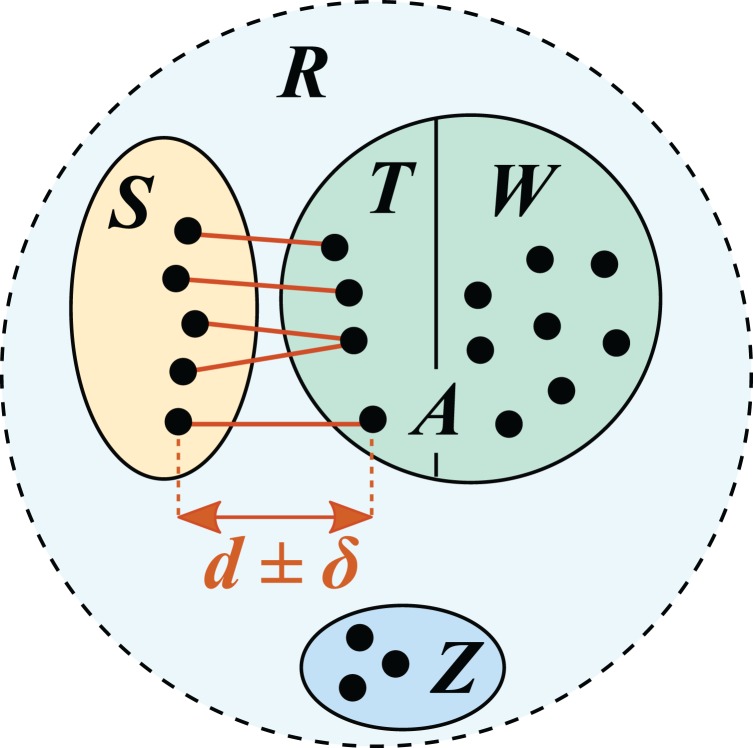
Test/training set construction for cross-validation by identity. *R* is a reference database, which is divided into disjoint subsets *S*, *T*, *W* and *Z*. *S* is the test set; the training set is *A* = *T* ∪ *W*. *T* is the set of top hits for sequences in *S*, which are constrained to be in the range *d* ± *δ* where *δ* specifies the maximum allowed deviation from the desired identity *d*.

In this work, I describe a new benchmark strategy, *cross-validation by identity* (CVI), which explicitly models varying distances between query sequences and reference sequences. A reference with known taxonomies is split into test and training sets such that for all test sequences, the most similar training sequence has a given identity (*top hit identity*, *d*), e.g., *d* = 97% (Fig. 1). This is repeated for different identities, enabling assessment of prediction accuracy at varying distances from the reference. For example, high accuracy at family rank is expected for query sequences having 100% identity with the reference database, but lower accuracy at 90%; these expectations can be validated by test/training pairs at 100% and 90% identities, respectively. Query sequences belonging to novel taxa, i.e., taxa not found in the reference, are modeled in test/training pairs with *d* < 100%. For example, most pairs of 16S rRNA sequences in a given genus have ≥95% identity (Yarza et al., 2014). Therefore, with *d* = 90% most test sequences will belong to genera which are absent from the training set, and with *d* = 95% there will be a mix of present and absent genera. Thus, novel taxa arise naturally by construction of the test/training pairs, and the frequency of novel and known taxa at each rank is determined by identity rather than taxonomy. Previous strategies such as LCO enable measurement of accuracy assuming it is known whether or not a given rank is novel, but it is not possible to determine novelty independently of taxonomy prediction and it is therefore not possible to extrapolate to accuracy in practice. By contrast, CVI enables measurement of prediction accuracy for all ranks at any given identity. Identity can be measured independently of taxonomy, enabling accuracy in practice to be estimated.

### Availability

Benchmark data, code and results are available at https://drive5.com/taxxi, which has been deposited in GitHub (https://github.com/rcedgar/taxxi).

## Methods

### Reference data

I used the NCBI BLAST 16S rRNA database (BLAST16S) (Sayers et al., 2012), downloaded July 1, 2017, the RDP 16S rRNA training set v16 (RDP16S) and the Warcup fungal ITS training set v2 (WITS) (Deshpande et al., 2015). The sequences and taxonomy annotations for these databases were mostly obtained from authoritatively named isolate strains. While there could be some errors in the taxonomy annotations, I pragmatically considered them to be authoritative and used them as truth standards for the benchmark tests. BLAST16S and the RDP 16S rRNA training set have highly uneven numbers of sequences per genus. For example, ∼40% (950/2,273) of the genera in BLAST16S have only a single sequence while the most abundant genus, *Streptomyces*, has 1,162 sequences, more than all singletons combined. To investigate the effects of uneven representation and create a more balanced reference, I created a subset (BLAST16S/10) by imposing a maximum of 10 sequences per genus; sequences were discarded at random as needed to meet this constraint. I also considered two larger databases: the subset of Greengenes clustered at 97% identity (GG97) which is the default 16S rRNA reference database in QIIME v1, and UNITE (Kõljalg et al., 2013). GG97 and UNITE were not used as truth standards because most of their taxonomy annotations are computational or manual predictions. To investigate prediction performance with shorter sequences, I extracted the V4 and V3–V5 segments from BLAST16S and BLAST16S/10 using V4 primer sequences from Kozich et al. (2013) and V3–V5 primer sequences from Methé et al. (2012). Sequence error was not modeled because state-of-the-art methods are able to extract highly accurate sequences from noisy next-generation reads (Edgar, 2013; Callahan et al., 2016).

### Sequences from in vivo samples

To investigate sequences from samples collected in vivo, I chose four recent datasets (Kozich et al., 2013; Wang et al., 2015; Bernstein et al., 2017; Howard, Bell & Kao-Kniffin, 2017) (Table 1). Human gut is a well-studied environment which should therefore have relatively high coverage in reference databases. Soil contains highly diverse communities of microbes which are often difficult to culture (Handelsman et al., 1998) and should therefore have lower coverage, presenting a more difficult challenge for taxonomy prediction. The hypersaline microbial mat was chosen as an example of a less studied environment which is also likely to have low coverage.

**Table 1:** Environmental datasets.

Environment	Tag	SRA accession	Reference
Human gut	V4	PRJEB16057	Wang et al. (2015)
Microbial mat	V4	PRJEB14970	Bernstein et al. (2017)
Soil	V4	(not deposited)	Kozich et al. (2013)
Soil	ITS	PRJNA378299	Howard, Bell & Kao-Kniffin (2017)

**Notes:**

Four datasets were chosen as shown in the table. The reads for Kozich et al. (2013) were downloaded from the authors’ web site (http://www.mothur.org/MiSeqDevelopmentData/, accessed 1st March 2017).

### Lowest common rank

The *lowest common rank* (LCR) of two sequences is the lowest rank where both have the same taxon name. For example, *Enterococcus avium* and *Pilobacter termitis* belong to different genera in the *Enterococcaceae* family, and their LCR is therefore family. The identity of a pair of sequences is an approximate guide to their LCR. For example, if their 16S rRNA identity is 92%, it is a reasonable guess that their LCR is family. With high confidence, the identity is too low for them to belong to the same species, and it is almost certain that the LCR is below phylum. The degree of certainty can be quantified by the probability that the LCR of a pair of sequences is a particular rank (e.g., family) given their pair-wise sequence identity (e.g., 92%). This probability depends on how sequences are selected, which can be specified by a frequency distribution over possible sequences. More formally, suppose two sequences are selected from distribution ***D*** and have identity *d*, then the probability that their LCR is *r* is written as *P*(LCR = *r* | ***D***, *d*). Given a reference database ***R***, a natural choice of distribution is random selection of sequences from ***R*** with replacement, denoted ***D***(***R***). For each authoritative taxonomy reference database ***R***, I calculated *P*(LCR = *r* | ***D***(***R***), *d*) for each rank as a function of *d* as follows (Fig. S1). The set of ranks is defined by the ranks which are annotated in the reference database; a rank variable such as *r* is an element of this set. Note that *r* is a rank, e.g., phylum, not a specific taxon at this rank. For BLAST16S and WITS, the set of ranks is {domain, phylum … species}, while for RDP16S the lowest annotated rank is genus. Comparisons between ranks are determined by domain > phylum > class > order > family > genus > species. I calculated *d* for each pair of sequences in ***R*** as the integer-rounded sequence identity according to USEARCH (Edgar, 2010). Let *M_d_* be the total number of pairs with identity *d*, and for each rank *r* let *m_d,r_* be the number of pairs with identity *d* for which LCR = *r*, then

(1) $$P\left( {{\rm{LCR}} = r\;|\;{\boldsymbol {D}}\left( {\boldsymbol{R}} \right),\,d} \right)\, = \,{m_{d,r}}/{M_d}.$$

A pair of sequences have the same name at rank *r* (i.e., belong to the same taxon at rank *r*) if and only if LCR ≤ *r*. For example, if species is the lowest rank, then they belong to the same genus if and only if LCR = genus or LCR = species. In general, the probability that the pair has a *common rank* (CR), i.e., the same name at rank *r*, is

(2) $${P_{\rm CR}}\left( r \right) = {\Sigma _{s \le {\rm{}}r}}P\left( {{\rm{LCR}} = s} \right).$$

The *most probable lowest common rank* (MLR) for a pair of sequences with identity *d* is defined as the LCR with highest probability,

(3) $${\rm{MLR}}\left( d \right) = argmax\left( r \right)P\left( {{\rm{LCR}} = r\;|\;d} \right).$$

If a pair of sequences has identity *d*, then the most likely inference is that they have the same name at rank MLR(*d*) and above, and different names at lower ranks. More formally, if LCR is regarded as a discrete random variable, then the expected value (Ross, 2007) of LCR given *d* is MLR(*d*). MLRs can be summarized by giving the *rank identity threshold* (RIT) for each rank *r*, defined as the minimum identity for which MLR(*d*) = *r*. For example, if MLR(100) = species, MLR(99) = genus, MLR(98) = genus … MLR(94) = genus and MLR(93) = family, then RIT(species) = 100 and RIT(genus) = 94. This is a principled method for calculating identity thresholds without clustering, noting that different clustering criteria such as single-, average- and complete-linkage give different thresholds (Sun et al., 2012; Edgar, 2018a).

### Top-hit identity distribution

For a given query set, e.g., Operational Taxonomic Unit (OTU) sequences from a sequencing experiment, distances from a reference database can be summarized by the *top-hit identity distribution* (THID). To obtain a THID, I calculated integer-rounded top-hit identities using USEARCH and displayed the results as a histogram showing the number of OTUs (*N_d_*) with each top-hit identity (*d*). This histogram visualizes the coverage of the query set by the reference database. A histogram bar is colored according to the MLR for its identity. For example, if the MLR is family, then the most probable inference is that the genus is novel for an OTU at that identity. While this inference is uncertain in any given case, the numbers of OTUs having higher and lower LCRs at that identity should tend to average out so that the overall distribution gives an informative, but still approximate, indication of the number of OTUs belonging to novel taxa at each rank. The THID for a L1O cross-validation test was determined by calculating the identity of the nearest neighbor for each reference sequence using USEARCH. For other cross-validation tests, it was obtained as the identity distribution of test/training set pairs. A cross-validation test is not realistic if its THID is substantially different from THIDs of query sets encountered in practice.

### Estimating the number of known and novel OTUs

The number of known and novel OTUs with respect to a reference database can be estimated from the common rank probabilities and THID. Consider a toy example where there are 100 OTUs, all of which have 97% identity, and suppose that *P*_CR_(species | *d* = 97%) = 0.7 and *P*_CR_(genus | *d* = 97%) = 1.0. Then the best estimate is that 70 OTUs belong to a known species and 30 OTUs belong to a novel species. In general, if the probability that rank *r* is known is *P*_CR_(*r*), then the probability that it is novel is 1−*P*_CR_(*r*), and the estimated number of known OTUs *K^est^_d_*(*r*) and novel OTUs *L^est^_d_*(*r*) at identity *d* are:

(4) $${K^{est}}_d\left(r \right) = {P_{\rm CR}}\left({r\;|\;d} \right){N_d},$$

(5) $${L^{est}}_d\left(r \right) = {\rm{ }}\left({1-{P_{\rm CR}}\left({r\;|\;d} \right)} \right){N_d}.$$

The estimated total number of known and novel OTUs at rank *r* can then be calculated by summing over identities:

(6) $${K^{est}}\left(r \right) = {\Sigma _d}\,{K^{est}}_d\left(r \right),$$

(7) $${L^{est}}\left(r \right) = {\Sigma _d}\,{L^{est}}_d\left(r \right).$$

### Cross-validation by identity

A test/training set pair was constructed by choosing a value *d* for the top-hit identity and dividing the reference database (***R***) such that the identity of the top training hit for every test sequence is approximately *d*. I chose the following values for *d* as representative of top-hit identities encountered in practice: 100, 99, 97, 95 and 90%. With *d* = 100%, the test and training sets are both identical to ***R***. For each *d* < 100%, ***R*** was divided into four disjoint subsets *S*, *T, W* and *Z* (Fig. 1). *S* is the test set and *A* = *T* ∪ *W* is the training set. *T* is the set of top hits for *S*, which are constrained to have identities in the range *d* ± *δ* where *δ* specifies the maximum allowed deviation from *d*. With *d* = 100, *δ* = 0 because the top-hit identity constraint is trivially satisfied by using the complete reference database as both the test and training set. With *d* < 100%, setting *δ* > 0 makes it easier to satisfy the identity constraint, enabling larger test sets to be constructed. I used *δ* = 1% for *d* = 90% and *δ* = 0.5% for *d* = 99, 97 and 95%. *W* contains sequences having identities <*d* with all members of *S. Z* contains sequences that are discarded because they would violate the top-hit identity constraint if they were assigned to *S*, *T* or *W*. These subsets were constructed by a greedy algorithm which first attempts to maximize the number of sequences in *S* (which also determines *T*), then assigns the remaining sequences to *W* if possible or *Z* otherwise (R. Edgar, 2018, unpublished data). The details of the greedy algorithm are unimportant here as it is straightforward to verify that the identity constraints were correctly satisfied by aligning a test set to its training set; all top hits should be in the range *d* ± *δ*.

### CVI performance metrics

In typical testing of a classifier algorithm, each prediction is categorized as a true positive, true negative, false positive or false negative (Fawcett, 2006). However, taxonomy prediction is not typical because categories are hierarchical and reference data is sparse in two respects: most sequences are not known, and most known sequences do not have authoritative classifications (Yarza et al., 2014). As a result, the most challenging step in taxonomy prediction is determining the LCR between the query sequence and an authoritative reference, a task which has no analog in a typical classification problem. This motivates distinguishing *over-classification errors*, where too many ranks are predicted, *under-classification errors*, where too few ranks are predicted, and *misclassification errors* where a known name is incorrectly predicted (Edgar, 2014, 2016). The algorithms considered here do not explicitly make predictions that a rank is not known. A rank which is not predicted (left blank, or below a confidence threshold) should not be interpreted as a prediction that the rank is not known because it could also be a known rank where the name is unresolved, e.g., because two named species have the same sequence. The usual definition of a true negative is therefore not applicable here. With these considerations in mind, I defined performance metrics for a given rank as follows. Let *N* be the number of sequences in the test set *S*, *K* be the number of sequences in *S* with known names, i.e., names which are present in the training set *A*, and *L* = *N* – *K* be the number of novel test sequences, i.e., sequences in *S* with names that are not present in *A*. Let *TP* be the number of names which are correctly predicted, *MC* the number of misclassification errors, *OC* the number of over-classification errors, and *UC* the number under-classification errors. The rate for each type of error is defined as the number of errors divided by the number of opportunities to make that error: *OCR = OC*/*L* (over-classification rate), *UCR* = *UC*/*K* (under-classification rate) and *MCR* = *MC/K* (misclassification rate). The true positive rate is *TPR* = *TP*/*K*, i.e., the number of correct names divided by the number of opportunities to correctly predict a name. *Accuracy* is calculated as *Acc* = *TP*/(*K* + *OC*), i.e., the number of correct predictions divided by the number of predictions for which correctness can be determined. The value of accuracy ranges from a maximum of one (no errors) to a minimum of zero (no correct predictions). It accounts for all test sequences with names that are known and/or predicted by the algorithm. Names which are not known and not predicted are excluded because correctness cannot be determined in these cases (see discussion of true negatives above). A metric is not reported if the denominator was less than 10, i.e., at least 10 test cases were required to calculate a rate. For each rank, I calculated the mean values of the metrics over all test/training pairs for all values of the top-hit identity (*d*). These are designated by the prefix *Avg*; for example, *AvgAcc* is the mean accuracy for a given rank, say genus. The averaged metrics should be realistic in the sense that their values will fall within the range typically encountered in practice, while noting that the THID for a given dataset may be quite different from the distribution which is implicitly modeled by averaging, i.e., equal numbers of query sequences with *d* = 100, 99, 97, 95 and 90 (see Supplemental Information 3 for further discussion). However, no single metric can reliably predict performance on an arbitrary query set because THIDs vary, prediction performance varies with identity, and some choice of weights must be made to calculate a summary metric. Equal weighting is a reasonable choice considering the wide variety of identity distributions that are encountered in practice.

### L1O testing

I also performed L1O testing on the same reference databases for comparison with CVI. With L1O, every test sequence *Q* has a different training database constructed by deleting *Q* from the reference. The four reference databases used for CVI testing (BLAST16S/10, BLAST16S/10-V4, BLAST16S/10-V35 and WITS) contain a total of 136,178 sequences and L1O testing therefore naively requires executing a method on ∼10^5^ distinct test/training pairs, which is impractical for most methods with the computational resources available to a typical investigator. Storing the test/training data in FASTA format would require ∼1Tb, and while this data could be constructed on the fly, some methods require minutes or hours for training on a new database plus generating a prediction for one query, and the total execution time would be prohibitive. I therefore re-implemented RDP, Q1 and Q2_VS in USEARCH with optimizations enabling L1O in a single pass through the reference by masking out the query sequence at run-time (-*self* option). Here, I refer to these re-implemented methods as NBC, CT1 (consensus taxonomy per QIIME v1) and CT2 (consensus taxonomy per QIIME v2), respectively. The native Java implementation of RDP supports a L1O feature, but it does not impose a bootstrap cutoff and does not report individual predictions, so it was necessary to re-implement the algorithm to enable L1O testing with a cutoff and calculation of accuracy metrics not supported by the RDP code. SINTAX has a query exclusion option and was also included in L1O testing. Other methods were not re-implemented due to the difficulty of writing the code and/or because the training algorithm was not amenable to optimized masking of the query. CVI results are reported for the re-implemented methods for comparison with the original packages (complete results at the TAXXI web site, https://drive5.com/taxxi); these show that the corresponding USEARCH commands give comparable results with variations that are consistent with random fluctuations in bootstrapping (NBC vs. RDP) or the use of *k*-mer search algorithms with different optimizations and tie-breaking strategies (CT1 vs. Q1 and CT2 vs. Q2_VS).

#### The Acc_RDP_ accuracy metric

The L1O testing reported in Wang et al. (2007) and Deshpande et al. (2015) used a measure of accuracy (*Acc*_RDP_) which is calculated as the number of correctly predicted names divided by the total number of query sequences,

(8) $$Ac{c_{RDP}} = \,TP/N.$$

A novel name cannot be correctly predicted, so the maximum value of *Acc*_RDP_ is *K*/*N*, where *K* is the number of sequences with known names. If there are novel names, then *K* < *N* and the maximum possible value of *Acc*_RDP_ is less than one, in contrast to conventional measures of accuracy which have a maximum achievable value of one for an ideal algorithm. With L1O testing, a novel name arises from a singleton taxon in the reference, and the maximum possible *Acc*_RDP_ is therefore the fraction of reference sequences having non-singleton names at the tested rank. Values of *Acc*_RDP_ are reported for comparison with previous work.

### Top-hit methods

A *top-hit classifier* predicts that the query taxonomy is identical to the taxonomy of the top hit. This is a simple strategy with an obvious limitation: novel taxa will always be over-classified if any hits are found. A top-hit classifier provides a baseline standard of comparison for more complicated methods. I tested top-hit methods based on three different search algorithms: (1) the USEARCH algorithm, using identity according to global alignment, (2) BLAST, using identity of the local alignment, and (3) the USEARCH algorithm using *k*-mer similarity without making alignments. I call these methods TOP, BTOP and KTOP, respectively. KTOP finds the reference sequence with most unique eight-mers in common with the query sequence, which is essentially equivalent to SINTAX with zero bootstrap. For all three methods, ties for highest identity or *k*-mer similarity were resolved by using the first hit reported by the software.

### Tested prediction methods

I tested the methods shown in Table 2. Three bootstrap thresholds were used for RDP: 80%, as recommended by its authors, 50% because this is the default in QIIME v1, and zero because this was the threshold used in the L1O testing reported in Wang et al. (2007) and Deshpande et al. (2015). The same thresholds were used for SINTAX for comparison. GAST, MEGAN, PROTAX, 16Sclassifier, RaxML and pplacer were not tested due to implementation issues (Supplemental Information 1).

**Table 2:** Tested taxonomy prediction methods.

Method	Software	Algorithm reference
BLCA	blca May 11, 2017	Gao et al. (2017)
BTOP	blastn v2.2.31+	Altschul et al. (1990)
CT1	usearch v11.0	Re-implements Q1
CT2	usearch v11.0	Re-implements Q2_VS
KNN	mothur v1.39.5	(Unpublished)
KTOP	usearch v11.0	(This paper)
Metaxa2	Metaxa 2.2-beta9	Bengtsson-Palme et al. (2015)
Microclass	microclass v1.1	Liland, Vinje & Snipen (2017)
NBC	usearch v11.0	Re-implements RDP
Q1	QIIME v1.9	(Unpublished)
Q2_BLAST	QIIME v2.2017.10	Bokulich et al. (2017)
Q2_SK	QIIME v2.2017.10	Bokulich et al. (2017)
Q2_VS	QIIME v2.2017.10	Bokulich et al. (2017)
RDP	Classifier v.2.4	Wang et al. (2007)
SINTAX	usearch v11.0	Edgar (2016)
SPINGO	SPINGO v1.3	Allard et al. (2015)
TOP	usearch v11.0	(This paper)

**Notes:**

Default options were used for all methods unless otherwise stated. A beta version of Metaxa2 was used because this was the only build supporting for a user-defined database at the time this work was performed. Three methods (RDP, Q1 and Q2_VS) were re-implemented in USEARCH (NBC, CT1 and CT2, respectively) with optimizations to enable L1O testing.

## Results

### Top-hit identity distributions

**Figure 2:**
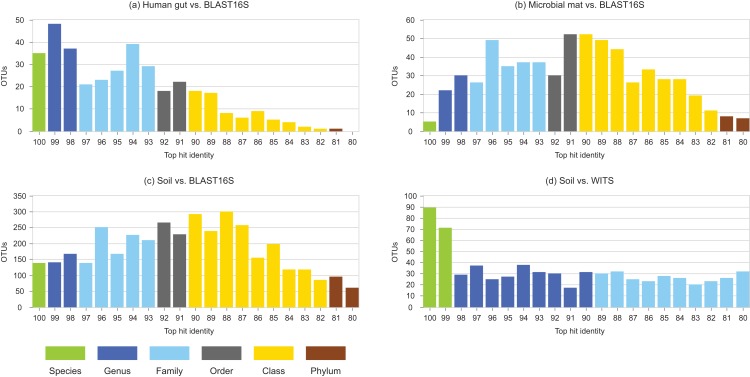
Top-hit identity distributions using BLAST16S or WITS as a reference. Datasets are specified in Table 1. Histogram (A) is human gut vs. BLAST16S, (B) is soil vs. BLAST16S, (C) is microbial mat vs. BLAST16S and (D) is soil vs. WITS. Histogram bars are colored according to the most probable lowest common rank at each identity. For example, at 95% identity, the most probable LCR for BLAST16S is family (light blue). The most probable inference for an OTU at this identity is that it belongs to a novel genus in a known family. This is approximate in any given case, but should tend to average over a set of OTUs so that the distribution gives an indication of the amount of novelty at each rank.

**Figure 3:**
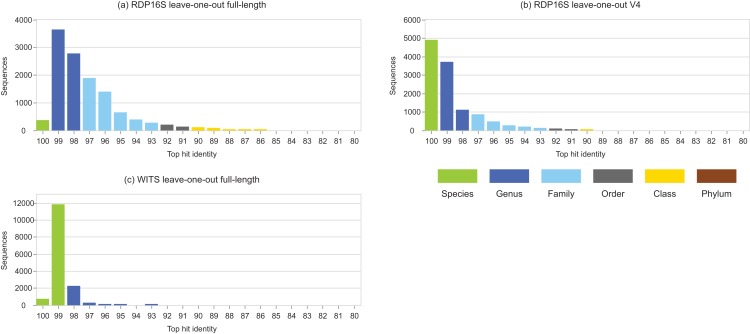
Top-hit identity distributions for RDP leave-one-out tests. Panel (A) is the top-hit identity distribution for the full-length RDP 16S rRNA training set, (B) for the V4 region of that training set, and (C) for WITS (the RDP Warcup training set). These distributions are strongly skewed toward higher identities compared to those in Fig. 2, showing that the leave-one-out tests are not realistic.

**Figure 4:**
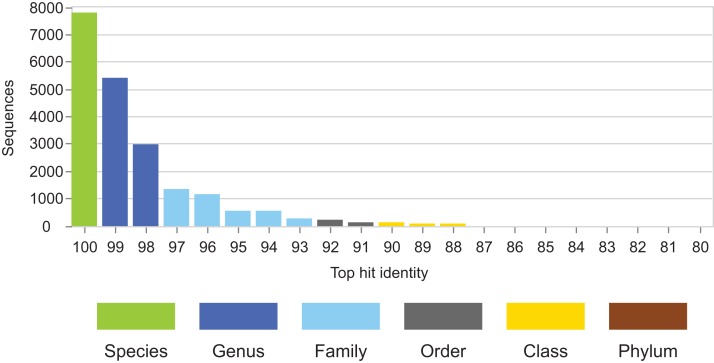
THID for dataset B from Bokulich et al. (2017). The top-hit identity distribution for dataset B from Bokulich et al. (2017), which has test/training sets for 10-fold cross-validation of V4 sequences from Greengenes. Most test sequences have 100% or 99% identity with the training sets, while in the environmental datasets (Fig. 2) most query sequences have ≤98% identity except for human gut. This test is therefore not realistic.

Identity distributions for the in vivo datasets using BLAST16S or WITS as references are shown in Fig. 2. All datasets exhibit broad distributions, showing that in practice, an algorithm using these references must often make predictions for query sequences with identities ≪100%. Distributions for the same datasets using GG97 and UNITE as references are shown in Fig. S2. These distributions are skewed more toward higher identities, as expected considering that these databases are much larger than the RDP training sets and should therefore have better coverage. Almost all OTUs in the human gut dataset have ≥98% identity with GG97, but with the other datasets many OTUs are observed to have lower identities with the larger databases, and in the soil ITS data a long tail is seen with many OTUs having <80% identity. Distributions for L1O tests on the RDP training sets are shown in Fig. 3. These distributions are very different from the in vivo datasets (Fig. 2), exhibiting strong skews toward high identities. This shows that the RDP L1O strategy is not realistic for assessing performance in practice. Taxonomies for high-identity queries are easier to predict, and the reported accuracy of RDP according to L1O testing (Wang et al., 2007; Deshpande et al., 2015) is therefore overestimated, especially for lower ranks. The THID for the 10-fold stratified cross-validation tests of Bokulich et al. (2017) are shown in Fig. 4 (dataset B) and Fig. S3 (dataset F). For the prokaryotic dataset (B), most test sequences have 99% or 100% identity to the training set, and this test is therefore not realistic. For fungal ITS (dataset F), the distribution is more realistic.

### CVI average accuracy for genus predictions

Complete results are available at the TAXXI web site, https://drive5.com/taxxi. Here, I describe some of the most informative results. The averaged accuracy of the tested methods for genus predictions (*AvgAcc*_genus_) on the BLAST16S/10 and WITS references are shown in Table 3. The BLAST16S/10 reference was selected as more realistic than BLAST16S because of its lower bias in sampling of sequences per genus (see Discussion). On the V4 region, the best methods have genus accuracies ∼50%, which improve to ∼60% on V3–V5 and ∼70% on full-length 16S rRNA. Rankings are seen to correlate between on the four tested segments (V4, V3–V5, full-length 16S rRNA and full-length ITS), but only approximately, and smaller differences in average accuracy should therefore be interpreted as benchmark artifacts rather than meaningful differences in algorithm performance. Most methods have very low accuracy on 16S rRNA sequences, especially KNN, with *AvgAcc*_genus_ ranging from 1.7% (V4) to 3% (full-length). The Q2_VS and Q2_BLAST methods also have very low accuracy on 16S rRNA, ranging from 16.1% (Q2_BLAST on V4) to 26.3% (Q2_VS on full-length sequences). All tested QIIME methods have average accuracy on 16S rRNA which is substantially less than RDP (excepting RDP50, which is a non-default option in QIIME v1). At the tested identities, higher accuracy is observed for all methods on ITS sequences, which reflects that the ITS region is less conserved than small-subunit rRNA (Thiéry et al., 2016). As a result, a test genus is usually present in the training set, and prediction is therefore easier than with 16S rRNA where genera are often absent at 95% and 90% identities. In practice, many ITS OTUs with identities <90% are observed (Fig. 2), and the *AvgAcc* metrics for ITS reported here therefore probably over-estimate accuracy in practice. This could be addressed by including lower identities in a future update of the benchmark.

**Table 3:** Average accuracy by CVI for genus on BLAST16S/10 and WITS.

Method	BLAST16S/10 V4	BLAST16S/10 V3–V5	BLAST 16S/10 full-length	WITS full-length
SINTAX50	50.3	59.3	67.7	87.0
RDP50	49.5	57.0	65.3	86.3
NBC50	49.4	56.9	65.3	86.4
BLCA	47.4	56.9	63.3	84.4
RDP80	47.3	58.4	68.4	82.5
NBC80	46.8	58.3	68.4	82.5
SPINGO	46.2	57.9	67.1	84.4
Microclass	45.0	54.2	63.4	87.4
BTOP	44.5	52.4	62.1	87.1
TOP	44.3	52.3	62.4	87.0
KTOP	44.2	52.6	62.7	86.7
SINTAX80	42.0	48.9	58.2	80.4
Q2_SK	38.7	45.6	57.9	85.4
Q1	38.3	45.1	53.8	84.5
CT1	37.7	45.7	55.4	84.0
Metaxa2	19.2	26.7	35.9	64.5
CT2	16.3	19.7	26.2	72.7
Q2_BLAST	16.1	19.1	23.5	69.9
Q2_VS	16.1	19.7	26.3	75.2
KNN	1.7	2.3	3.0	47.3

**Notes:**

Values are *AvgAcc*_genus_ expressed as percentages.

### CVI average metrics for V4 genus predictions

Averaged metrics for genus predictions on V4 sequences in BLAST16S/10 are shown in Table 4, with L1O accuracies for comparison. This shows that KNN has a very low true-positive rate (*AvgTPR*_genus_ = 1.7%) due to under-classification errors (*AvgUCR*_genus_ = 98.3%). Methods that achieve higher true positive rates have higher over- and misclassification rates. The true positive rate for RDP50 is 28% higher than RDP80 (65.6%/51.3%), but the over-classification rate is more than doubled (43.0%/14.8%) so that with RDP50, almost half of novel genera were incorrectly predicted to have known names. A similar trade-off is seen with SINTAX, showing that reducing the bootstrap threshold increases the numbers of both correct and incorrect predictions, as might be expected.

**Table 4:** Average metrics by CVI for genus predictions on the V4 segment of BLAST16S/10.

Method	AvgTPR_genus_	AvgUCR_genus_	AvgMCR_genus_	AvgOCR_genus_	L1OAcc_genus_	AvgAcc_genus_
SINTAX50	57.5	34.1	8.5	20.9	77.5	50.3
RDP50	65.6	18.0	16.4	43.0	–	49.5
NBC50	65.8	17.3	16.9	44.9	76.8	49.4
BLCA	54.6	40.3	5.1	16.7	–	47.4
RDP80	51.3	43.5	5.2	14.8	–	47.3
NBC80	50.8	43.9	5.3	15.3	70.1	46.8
Microclass	70.5	0.0	29.5	100.0	–	45.0
BTOP	70.7	0.0	29.3	100.0	–	44.5
TOP	69.5	0.0	30.5	100.0	75.8	44.3
KTOP	68.4	0.0	31.6	100.0	75.6	44.2
SINTAX80	43.8	55.1	1.1	7.3	67.0	42.0
Q2_SK	53.7	23.6	22.7	52.2	–	38.7
Q1	54.5	21.3	24.2	67.0	–	38.3
CT1	55.8	14.6	29.7	71.9	67.6	37.7
Metaxa2	21.3	75.1	3.6	10.2	–	19.2
CT2	19.5	67.6	12.9	27.7	39.7	16.3
Q2_BLAST	19.0	70.8	10.2	24.8	–	16.1
Q2_VS	19.3	67.4	13.3	26.9	–	16.1
KNN	1.7	98.3	0.0	0.1	–	1.7

**Notes:**

Values are averaged metric values expressed as percentages. *L1OAcc*_genus_ is calculated according to the CVI *AvgAcc* definition, not the *Acc*_RDP_ definition. See Table 6 and supplementary data for *Acc*_RDP_ values.

### Accuracy of species predictions

Average accuracy of species predictions is shown in Table 5. All tested methods have accuracies <20% on V4, ≤30% on full-length 16S rRNA and ≤35% for full-length ITS. These results are in sharp contrast to some previously reported measures of species prediction accuracy. For example, RDP was reported to have ∼85% accuracy for species predictions on WITS according to L1O testing (Deshpande et al., 2015), and the authors of SPINGO reported ∼90% accuracy of species predictions on V4 based on 10-fold cross-validation (Allard et al., 2015). However, correctly predicting ∼90% of species names for V4 metagenomic sequences is surely not achievable in practice by any algorithm using any current or future reference database because identical V4 sequences belong to different species with ≫10% probability, e.g., 63% probability according to BLAST16S and 37% according to BLASTS16S/10, calculated as *P*(LCR > species | BLAST16S-V4, 100%) = 0.63 and *P*(LCR > species | BLAST16S/10-V4, 100%) = 0.37, respectively.

**Table 5:** Average accuracy for species predictions.

Method	BLAST16S/10 V4	BLAST16S/10 V3–V5	BLAST16S/10 full-length	WITS full-length
SINTAX80	19.8	23.2	30.3	32.3
Microclass	18.5	21.2	24.7	35.1
NBC50	18.3	20.7	24.5	34.1
BTOP	18.2	21.1	24.6	35.3
TOP	18.0	21.1	24.7	35.2
KTOP	18.0	21.0	24.7	34.9
SINTAX50	17.9	22.3	27.7	35.2
RDP50	17.8	20.6	24.5	34.1
NBC80	17.4	20.9	25.4	33.6
RDP80	17.1	20.9	25.2	33.6
BLCA	16.9	21.3	24.9	34.7
SPINGO	16.8	21.3	25.9	35.0
Q2_SK	10.6	12.6	18.7	28.3
Q1	6.6	7.6	9.8	25.1
CT1	5.9	6.9	9.6	22.5
Metaxa2	5.6	10.4	6.8	1.0
Q2_VS	0.1	2.7	0.3	5.6
CT2	0.1	2.7	0.3	3.1
Q2_BLAST	0.1	0.4	0.2	0.3
KNN	0.0	0.0	0.0	0.0

**Notes:**

Values are *AvgAcc*_species_ expressed as percentages.

### Accuracy metrics for L1O tests

Leave-one-out performance metrics for genus predictions on the V4 region of BLAST16S/10 are shown in Table 6. Complete results are available at the TAXXI web site, https://drive5.com/taxxi. For NBC and SINTAX, the *Acc*_RDP_genus_ metric is highest at zero bootstrap threshold, reflecting that the only opportunities to make over-classification errors are singleton taxa, which are a small fraction of the reference sequences. As a result, increasing the true-positive rate by reducing the confidence threshold to zero (e.g., from *TPR*_genus_ = 72.6 for NBC80 to *TPR*_genus_ = 85.1 for NBC0) gives a higher value of *Acc*_RDP_ despite a large increase in the over-classification rate (from *OCR*_genus_ = 25.2 for NBC80 to *OCR*_genus_ = 100.0 for NBC0). It is noteworthy that KTOP, a simple *k*-mer top-hit method which will always over-classify, achieves higher *Acc*_RDP_genus_ than the NBC algorithm (*Acc*_RDP_genus_ = 75.6 for KTOP, compared to *Acc*_RDP_genus_ = 74.6 for NBC0 and *Acc*_RDP_genus_ = 63.6 for NBC80).

**Table 6:** Accuracy metrics by L1O for genus predictions on the V4 segment of BLAST16S/10.

Method	Acc_genus_	Acc_RDPgenus_	TPR_genus_	UCR_genus_	MCR_genus_	OCR_genus_
SINTAX50	77.5	71.3	81.3	13.8	4.9	35.4
NBC50	76.8	72.7	82.9	7.1	9.9	56.7
SINTAX0	76.0	76.0	86.7	0.0	13.3	100.0
TOP	75.8	75.8	86.5	0.0	13.5	100.0
KTOP	75.6	75.6	86.2	0.0	13.8	100.0
NBC0	74.6	74.6	85.1	0.0	14.9	100.0
NBC80	70.1	63.6	72.6	23.7	3.8	25.2
CT1	67.6	64.8	73.9	11.0	15.1	67.0
SINTAX80	67.0	60.1	68.5	30.0	1.5	15.9
CT2	39.7	35.8	40.9	48.9	10.2	21.1

**Notes:**

As Table 4 except that L1O was used instead of CVI. Note that accuracies are much higher according to L1O.

### Performance variation with identity

**Figure 5:**
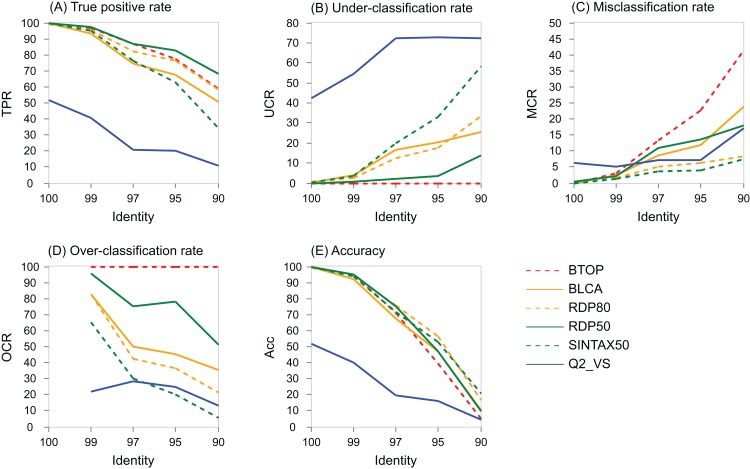
Performance metric variation with identity for genus predictions on BLAST16S/10. Performance metrics for genus predictions by some of the tested methods on the V4 region of BLAST16S/10 (*y* axis) plotted against the top-hit identity of test-training set pairs (*x* axis). Metrics are defined in the main text; (A) TPR (true-positive rate), (B) UCR (under-classification rate), (C) MCR (misclassification rate), (D) OCR (over-classification rate), and (E) Accuracy. Plots for other regions and databases are provided at the TAXXI web site.

The variation with identity of performance metrics for genus on the V4 region of BLAST16S/10 is shown for selected methods in Fig. 5 and on WITS in Fig. S4. Full results are available at the TAXXI web site, https://drive5.com/taxxi. As expected, the true positive rate and accuracy are highest at 100% identity and fall rapidly as identity decreases, while the under- and misclassification rates increase.

### *P*(LCR) as a function of identity

**Figure 6:**
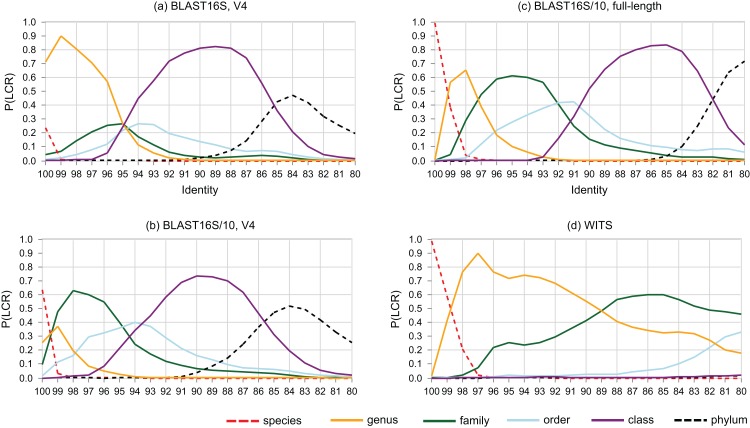
Lowest common rank probability as a function of identity. Lowest common rank probability for ranks from phylum to species on four reference databases: (A) the V4 region of BLAST16S, (B) the V4 region of BLAST16S/10, (C) full-length BLAST16S, and (D) WITS. The difference between BLAST16S and BLAST16S/10 at high identities is striking considering that they contain the same genera, differing only in the number of sequences per genus.

The probability that a pair of sequences has a given LCR is shown as a function of identity in Fig. 6. At lower ranks, striking differences are apparent between the V4 region of BLAST16S and BLAST16S/10. For example, *P*(LCR = genus | BLAST16S-V4, 97%) = 0.70 ≫ *P*(LCR = genus | BLAST16S/10-V4, 97%) = 0.09, and *P*(LCR = family | BLAST16S-V4, 97%) = 0.21 ≪ *P*(LCR = family | BLAST16S/10-V4, 97%) = 0.60. These references contain the same genera, differing only in the number of sequences for genera that are highly abundant in BLAST16S. Thus, reference databases containing the same taxa with different sampling biases can lead to different conclusions. For example, suppose a pair of V4 sequences has 97% identity. If the pair was randomly sampled from BLAST16S-V4, then the most likely inference is that they belong to different species in the same genus (LCR = genus with probability 0.7), but if they were sampled from BLAST16S/10-V4, they are more likely to belong to different genera in the same family (LCR = family with probability 0.6).

### Rank identity thresholds

Rank identity thresholds are given in Table 7. Some ranks have no threshold. For example, on BLAST16S/V4, the most probable LCR is genus for all identities ≥95%, so there is no species threshold, and on BLAST16S/V3–V5 the most probable LCR is class for identities 83–94% and genus for identities ≥95%, so there is no threshold for order, family or species. Note that if *r* is the most probable LCR, this does not necessarily imply that the LCR is probably *r*. For example, at 100% identity on BLAST16S-V4, the most probable LCR is genus, but *P*(LCR = genus |100%) = 0.49, so in fact the LCR is slightly more likely to be some other rank: species (*P* = 0.30), family (*P* = 0.19) or order (*P* = 0.03). The maximum LCR probability can be even lower, e.g., on BLAST16S-V35, *P*(LCR = class | 83%) = 0.39.

**Table 7:** Rank identity thresholds.

Reference	Segment	Species	Genus	Family	Order	Class
BLAST16S	Full-length	100	94	93	92	82
BLAST16S/10	Full-length	100	98	93	91	82
BLAST16S	V3–V5	–	95	–	–	83
BLAST16S/10	V3–V5	100	99	95	93	84
BLAST16S	V4	–	96	95	–	86
BLAST16S/10	V4	–	100	95	94	86
WITS	Full-length	99	90	–	–	–

**Notes:**

See main text for explanation of missing values.

### Estimated numbers of novel OTUs from in vivo datasets

The numbers of OTUs estimated to belong to novel taxa are shown in Table 8. Similar results are obtained using BLAST16S/10, which has reduced sampling bias, and the RDP 16S training set, which has strong sampling bias similar to BLAST16S. These results suggest that reference set bias does not have a large effect on this task.

**Table 8:** Numbers of OTUs estimated to belong to novel taxa.

Set	Tag	Species	Genus	Family	Order	Class	Phylum
(a) RDP training sets							
Gut	16S	–	222 (59.8%)	165 (44.5%)	49 (13.2%)	7 (1.9%)	0
Mat	16S	–	525 (78.6%)	454 (68.0%)	207 (31.0%)	95 (14.2%)	36 (5.4%)
Soil	16S	–	3233 (79.4%)	2812 (69.1%)	1348 (33.1%)	648 (15.9%)	268 (6.6%)
Soil	ITS	1828 (84.5%)	1271 (58.7%)	495 (22.9%)	44 (2.0%)	21 (1.0%)	0 (0.0%)
(b) BLAST16S/10							
Gut	16S	353 (95.1%)	296 (79.8%)	188 (50.7%)	110 (29.6%)	11 (3.0%)	0
Mat	16S	624 (93.4%)	594 (88.9%)	476 (71.3%)	358 (53.6%)	114 (17.1%)	35 (5.2%)
Soil	16S	3823 (93.9%)	3627 (89.1%)	2928 (71.9%)	2244 (55.1%)	769 (18.9%)	269 (6.6%)

**Notes:**

The number of novel OTUs for each rank was determined from the top-hit identity distribution and LCR probabilities as described in the main text. (a) shows results for the RDP 16S rRNA and ITS training sets, (b) for the BLAST16S/10 database.

## Discussion

### Identity as a predictor of rank

Sequence identity is sometimes used as an approximate indicator of rank (Stackebrandt & Goebel, 1994; Yarza et al., 2014). LCR probabilities give a new perspective. For example, Fig. 6 confirms the result of (Edgar, 2018a) that the commonly-used 97% threshold is a poor predictor that a pair of 16S rRNA sequences belong to the same species because *P*(LCR = species | 97%) is close to zero, with *P* = 0.001 on the V4 region of BLAST16S/10, *P* = 0.0004 on the V4 region of BLAST16S and *P* = 0.009 on full-length sequences in BLAST16S/10. Here, *P*(LCR = species) is equivalent to the *conspecific probability* of (Edgar, 2018a). LCR probabilities have the advantage of independence from clustering methods and cluster quality metrics, which give conflicting results for optimal threshold values (Edgar, 2018a). However, the BLAST16S and BLAST16S/10 databases contain the same genera, yet give substantially different LCR probabilities in the range *d* = 90–100%. This shows that LCR probabilities depend on taxon frequencies in addition to the set of known taxa, and consequently there can be no universally optimal identity thresholds or universally applicable LCR probabilities. This raises the related question of whether it is better to implement cross-validation tests using a highly biased reference database such as BLAST16S or a less biased reference such as BLAST16S/10. Reference databases are strongly biased toward taxa that are relevant to human health and can be cultured in the laboratory. While data obtained from some environments may have somewhat similar biases, e.g., human gut, other datasets will contain many taxa which are less studied and/or hard to culture, e.g., samples extracted from soil or an extremophile environment such as a hypersaline mat. It is therefore conservative to use a reference with reduced sampling bias. Otherwise, taxa that are strongly over-represented in the reference will tend to be over-represented in both the test and training subsets, causing unrealistically high accuracy estimates because a test sequence from an over-represented group is anomalously easy to predict due to the unusually large number of related sequences in the reference, and such sequences will be common in the test set and therefore contribute disproportionately to the measured accuracy. Consensus methods are particularly prone to this problem because taxa with high frequencies in the reference are implicitly considered to be more probable in the query dataset (Supplemental Information 2).

### Alternatives to identity as a predictor of taxonomy

Taxonomy is usually defined by characteristic traits. These traits are surely determined by many genes, and conservation of these genes surely correlate only approximately with conservation of a single marker sequence, placing a fundamental limit on prediction accuracy. It is well-recognized that identity of a marker sequence such as 16S rRNA or ITS is only an approximate guide to rank, raising the question of whether alternatives to identity can give more accurate predictions. Conceptually, the main evidence available is the taxonomy of the top hit and the similarity of the query to the top hit. Given that any measure of distance is at best only an approximate guide to taxonomy, identity is probably as good as any other measure of confidence or distance, noting as examples that *k*-mer similarity (KTOP) and alignment identity (TOP and BTOP) give very similar results, and KTOP achieves comparable or better performance to RDP by several metrics. In principle, placement into a phylogenetic tree uses more information by considering distances to parental groups and outgroups, but this approach involves several challenging steps: multiple alignment, tree inference, tree placement, resolving tree/taxonomy conflicts, and clade extension (Edgar, 2018b). I therefore doubt this approach would work better than the tested methods, but I was unable to verify this because to the best of my knowledge, there is no working implementation currently available in a published software package (Supplemental Information 1). Consensus methods such as those in QIIME and MEGAN use taxon frequencies in the top hits as an indicator of confidence, but relative frequencies are weighted by taxonomic bias and are therefore unreliable (Supplemental Information 2).

### Around 95% identity is a twilight zone for V4 taxonomy prediction

The LCR probabilities for V4 (Fig. 5) show that taxonomy is highly ambiguous at identities around 95%. With the V4 region of BLAST16S/10, the probabilities are approximately equal (*P* ∼ 0.2) that the LCR is genus, family, order or class. This suggests that at 95% identity, a reference database simply does not contain enough information to determine the rank of a V4 sequence, regardless of whether a prediction method explicitly uses an identity threshold. This is confirmed by low prediction accuracies at 95% identity where genus accuracy ranged from 36% (BLCA) to <1% (KNN). On the same reference, family accuracy ranged from 71% (RDP50) to 34% (KNN). Thus, 95% identity of V4 is a twilight zone for taxonomy prediction analogous to the twilight zone around 25% amino acid identity for proteins where sequence similarity becomes an unreliable predictor of homology and fold (Rost, 1999).

### Using a large database as a reference

The default reference database for QIIME v1 is GG97, a subset of Greengenes clustered at 97% identity. For QIIME v2, there is no default or recommended reference to the best of my knowledge, but SILVA and Greengenes were suggested as resources at the time of writing. Using a large database that includes environmental sequences will often have better coverage than a database containing mostly authoritatively named isolates, as shown by the identity distributions for GG97 in Fig. S2 which are skewed toward high identities compared with those for BLAST16S in Fig. 2. Prediction is more accurate at high identities, but the intrinsic error rate of a prediction algorithm will be compounded by the annotation error rate of the chosen reference database, which is surely higher for a database with mostly predicted annotations than a more authoritative reference such as RDP16S or BLAST16S, noting that I have previously estimated the error rates of the full RDP database to be ∼10% and SILVA and Greengenes to be ∼15% (Edgar, 2018b). Using a clustered reference database such as GG97 will tend to further degrade accuracy for query sequences that have identity >97% with a named isolate because the isolate sequence will probably be present in the full Greengenes database but will be absent from the GG97 subset unless it is the chosen representative for a cluster. More generally, for sequences belonging to well-known genera, identity with GG97 will usually be lower than an unclustered authoritative reference which would enable more accurate predictions, noting e.g., that *Acc*_genus_ for the better methods on V4 falls from ∼100% to ∼50% as identity falls from 100% to 97%.

### Toward realistic estimation of prediction accuracy

The TAXXI benchmark makes progress toward more realistic measurement of taxonomy prediction accuracy, while also demonstrating that a single metric cannot reliably predict performance on an arbitrary query set because identity distributions vary, and prediction performance varies with identity. Further development of the CVI approach can address this limitation to achieve more realistic estimates (Supplemental Information 3).

### Estimating metric errors and algorithm rankings

With randomized cross-validation tests such as LCO, *k*-fold and CVI, different metric values may be obtained if different pseudo-random numbers are used to generate test/training pairs. The variation of a metric (e.g., its standard deviation) can be measured by generating many randomized test/training pairs, and also the variation in algorithm rank order according to that metric, enabling a *P*-value to be calculated by a non-parametric method such as the Wilcoxon signed rank test. I did not attempt this in TAXXI for two reasons. First, it would be prohibitively expensive for the slower methods, increasing execution time by at least an order of magnitude (assuming that at least 10 pairs for each value of *d* are needed for a robust measurement of variation). Second, calculating *P*-values from randomization alone would give a misleading impression of statistical power because I would expect changing the taxonomic bias of the reference database to change metric values at least as much as randomization. This could be verified by measuring variations by randomization and also with different biases, e.g., by comparing results between BLAST16S and BLAST16S/10, though ideally more than two cases would be tested. This is a challenging project which I deferred to future work. These issues underscore that while performance metrics obtained by the current implementation of TAXII are more realistic than previous benchmarks, they should be interpreted only as rough guides to performance in practice, and TAXII therefore cannot provide definitive algorithm rankings.

### Directions for improvement

If no discernable improvement has been made in automated prediction since 2007, this suggests that a point of diminishing returns may have been reached in algorithm development. Adding more authoritative classifications to the reference databases would help, but the study and taxonomic classification of new isolates is a slow process undertaken by a small community of specialists which is unlikely to substantially increase the number of authoritatively classified microbes in the near future. An alternative solution for increasing the coverage of reference databases is to adopt standardized sequence-based classifications such as the species hypotheses in UNITE or Greengenes OTUs. The V4 hypervariable region is currently popular because its amplicon is a good match to fully-overlapped 2 × 250 reads, but the results reported here show that the taxonomic resolution of V4 is poor in practice. The same sequencing technology allows sequencing of V3–V4 and/or V4–V5 (Kozich et al., 2013), which give better taxonomic resolution by using partially-overlapping reads to sequence longer amplicons. Partial overlap need not result in higher sequence error rates because state-of-the art methods are able to extract highly accurate sequences from noisy reads (Edgar, 2013; Callahan et al., 2016; Edgar, 2017b), so I would recommend sequencing two hypervariable regions instead of one. In the near future, longer read lengths will enable amplicons such as V3–V5 as used by the Human Microbiome Project (Methé et al., 2012), and eventually full-length 16S rRNA, to be sequenced by low-cost, high-throughput next-generation machines. The particular implementation of CVI reported here (which I call TAXXI) considered V4, V3–V5 and full-length 16S rRNA and ITS sequences as a representative selection of marker segments and length. Somewhat different results would be obtained on different segments, but TAXXI results nevertheless enable a comparison of 16S rRNA markers of different lengths which should generalize approximately to other regions of similar lengths. Performance on regions of intermediate length, e.g., V4–V5, can be estimated by interpolation.

### Recommended choices of algorithm and reference

It is clear from the results reported here that taxonomy prediction methods cannot be definitively ranked according to their accuracy using TAXXI or previous benchmark tests. However, some trends are apparent which can guide a biologist toward an appropriate method for addressing a given research question. In most cases, the highest true-positive rates are achieved by the simple top-hit classifiers (see e.g., Table 4). This shows, as might be expected, that the true name of a known rank is usually present in the top hit annotation, and there is no indication that more complicated methods achieve any improvement in this respect by successfully identifying anomalous cases where a taxon is known but has a lower identity than the top hit. This shows that the biggest challenge in algorithm design can be framed as predicting the LCR, or, equivalently, deciding how many ranks of the taxonomy in the top hit should be deleted in the prediction. Most methods incorporate thresholds which determine the lowest rank to predict using a measure such as identity (MEGAN, Metaxa2), bootstrap (RDP, SINTAX, Q2_SK), posterior probability (BLCA), and/or frequency in the top hits (MEGAN, Q1, Q2_BLAST and Q2_VS). Adjusting a threshold necessarily makes a trade-off between true positives and false positives, because more true positives can be obtained only at the expense of including more false positives, reflecting the fact that taxonomy correlates only approximately with any measure of sequence similarity. Thus, different thresholds may be appropriate in different studies depending on whether false negatives or false positives are more important. This perspective also explains why using different thresholds, e.g., RDP50 and RDP80, often changes the CVI accuracy by only a small amount: the lower false positive rate of RDP80 is approximately balanced by its higher false negative rate so that neither is clearly superior to the other. A similar effect is seen with SINTAX and would presumably be seen in other methods with user-settable thresholds, though this was not tested here. A given threshold results in different trade-offs on different datasets, so it is not possible to derive a single best threshold by specifying a desired balance between different types of error. For example, SINTAX50 has the highest CVI accuracy of all methods for genus on the V4 region of BLAST16S/10, where it has 20% higher accuracy than SINTAX80, but for species predictions on the same dataset the roles are reversed: SINTAX80 has the highest accuracy of all methods, 11% higher than SINTAX50. Therefore, the 80% bootstrap cutoff which has been recommended for RDP and SINTAX is reasonable, but not clearly better than 50% or some other choice. The bootstrap methods SINTAX and RDP have comparable performance overall; neither is systematically better at the tested bootstrap values. SPINGO is also a bootstrap method, but it achieves no discernable improvement and predicts only family, genus and species. BLCA achieves comparable performance to bootstrap methods on most tests, but is much slower due to its use of BLAST. Microclass results are very similar to top-hit methods. Consensus methods are inferior to bootstrap methods on most tests, and KNN is clearly worse. Q2_SK is inferior to RDP and SINTAX on most tests. None of the newer algorithms is discernably better than the RDP Classifier overall, suggesting that little, if any, meaningful improvement in accuracy has been achieved since 2007. With these considerations in mind, and those of *Using a large database as a reference* above, I would suggest that for most prediction tasks encountered in practice, a bootstrap method should be used with an authoritative reference such as an RDP training set or the BLAST 16S rRNA database rather than SILVA or Greengenes. The bootstrap threshold should be chosen according to the marker sequence (e.g., V4) and goals of a study (e.g., considering whether true positives or false positives are more important at low ranks) by consulting tables of CVI metrics, which currently provide the best available indication of the trade-off between true positives and errors.

## Supplemental Information

10.7717/peerj.4652/supp-1 Supplemental Information 1 Implementation issues with methods that were not tested.

10.7717/peerj.4652/supp-2 Supplemental Information 2 Scenarios where concensus methods fail.

10.7717/peerj.4652/supp-3 Supplemental Information 3 Towards realistic estimation of prediction accuracy.

10.7717/peerj.4652/supp-4 Supplemental Information 4 Method for calculating LCR probabilities from a reference database. Simple example illustrating the method for calculating LCR probabilities. An all-vs-all distance matrix is constructed containing pair-wise sequence identities. Here, there are three different identities indicated by colors: green = 100%, yellow = 95% and orange = 90%. The lowest common rank (LCR) is determined for each pair by comparing taxonomy annotations. In this example, ranks are species (S), genus (G) and family (F). For each identity, the corresponding set of pairs is identified. There are three such sets, one for each of the three identities. For a given identity, the LCR frequency for a rank is calculated as the fraction of pairs having that rank. For example, there are eight pairs with 90% identity. Of these, there are no pairs with LCR = species, two pairs with LCR = genus, and six with LCR = family, so P(S|90%) = 0/8, P(G|90) = 2/8 and P(F|90) = 6/8.

10.7717/peerj.4652/supp-5 Supplemental Information 5 Top-hit identity distributions using GG97 or UNITE as a reference. Histogram (a) is human gut vs. GG97, (b) is soil vs. GG97, (c) is microbial mat vs. GG97 and (d) is soil vs. UNITE.

10.7717/peerj.4652/supp-6 Supplemental Information 6 Top-hit identity distribution for Bokulich et al. (2017) dataset F (fungal ITS). Histogram bars are colored according to the most probable lowest common rank.

10.7717/peerj.4652/supp-7 Supplemental Information 7 Performance metrics variation with identity for genus predictions on WITS. Performance metrics for genus predictions by some of the tested methods on WITS (*y* axis) plotted against the top-hit identity of test-training set pairs (*x* axis). Metrics are defined in the main text; (a) *OCR* (over-classification rate), (b) *UCR* (under-classification rate), (c) *MCR* (misclassification rate), (d) *Acc* (accuracy) and (e) *TPR* (true-positive rate).

## Additional Information and Declarations

### Data Availability

The following information was supplied regarding data availability: GitHub: https://github.com/rcedgar/taxxi.
