# Supplementary material for: Accuracy of taxonomy prediction for 16S rRNA and fungal ITS sequences
Source: PeerJ. 2018 Apr 18;6:e4652. doi: 10.7717/peerj.4652 (PMC5910792; doi:10.7717/peerj.4652)
Supplement: Supplemental Information 1 [file peerj-06-4652-s001.pdf]

Accuracy of taxonomy prediction for 16S rRNA and fungal ITS sequences

Robert C. Edgar

Supplementary Note 1

GAST, MEGAN, PROTAX, RaxML and pplacer were not tested because they do not provide complete solutions for predicting taxonomy. With these methods, users must choose parameters for which no recommended defaults or tuning procedures are documented. User-defined parameters for MEGAN include an identity threshold for each rank and the minimum fraction of BLAST hits required for a consensus name to be assigned to a rank. For RaxML and pplacer, users must provide multiple alignments and trees for the reference sequences. Prediction performance will therefore depend on the accuracy of the alignment and tree inference methods in addition to the tree-placement algorithm. With ITS sequences, robust multiple alignment is only possible for closely related taxa (Hibbett *et al.*, 2016). Also, tree placement is not sufficient for prediction because a post-processing algorithm is required to resolve ambiguities and mismatches between the taxonomy and the tree (Edgar, 2018). No such algorithm was supported by the versions of pplacer (v1.1) and RaxML (v8.0) available at the time this work was performed. Tax2tree provides a possible solution for post- processing, but I was unable to run the software and a request for support was not answered. The 16Sclassifier method was not tested because it does not support user-supplied reference databases.

References

Edgar RC. (2018). Taxonomy annotation errors in 16S rRNA and fungal ITS sequence databases.

Hibbett D, Abarenkov K, Kõljalg U, Öpik M, Chai B, Cole J, Wang Q, Crous P, Robert V, Helgason T, Herr JR, Kirk P, Lueschow S, O'Donnell K, Nilsson RH, Oono R, Schoch C, Smyth C, Walker DM, Porras-Alfaro A, Taylor JW, Geiser DM. (2016). Sequence-based classification and identification of Fungi. *Mycologia* **108**: 1049–1068.
