# Supplementary material for: Accuracy of taxonomy prediction for 16S rRNA and fungal ITS sequences
Source: PeerJ. 2018 Apr 18;6:e4652. doi: 10.7717/peerj.4652 (PMC5910792; doi:10.7717/peerj.4652)
Supplement: Supplemental Information 2 [file peerj-06-4652-s002.pdf]

Accuracy of taxonomy prediction for 16S rRNA and fungal ITS sequences

Robert C. Edgar

Supplementary Note 2: Scenarios where consensus methods fail

The QIIME methods Q1, Q2_VS and Q2_BLAST use a consensus approach where a rank is predicted if a simple majority (i.e., more than half) of the top few hits agree on the name; otherwise, no name is predicted for that rank. MEGAN and Metaxa2 use consensus combined with identity thresholds. With Q1, the top three hits are included; with Q2_VS and Q2_BLAST, the top ten hits are included. Consider the example in Fig. SN3.6, where F is a known family containing genera G and H, J is a genus in a different family and the query sequence Q belongs to a novel family that is not present in the reference. The closest reference sequences to Q are a, b, c and d which are approximately equidistant from Q. Q1 keeps only three of the top hits, which gives a 2/3 majority to G or H. Here, Q1 will over-predict the family as F and over-predict the genus as G or H. Q2_VS and Q2_BLAST keep the top 10 hits, so the more distant reference sequences e, f and g will also be included. When a–g are included, there is no majority consensus, so no family or genus will be predicted. This is correct because Q belongs to a novel genus in a novel family. However, if b and d are missing from the reference, then there will be a 3/5 majority for the family and genus of J, and both ranks will therefore be over-classified. In this last scenario, G and H have higher identity than J, but Q is nevertheless predicted to belong to J because it has more reference sequences. Thus, consensus methods do not consider sequence similarity after a fixed number of top hits have been selected, and effectively assume that taxa which are more

abundant in the reference are more probable in the query. These examples illustrate that for any choice of parameters such as the number of top hits to include and the minimum consensus fraction, it is straightforward to find realistic situations where incorrect, and sometimes counter-intuitive, predictions will be made by a consensus method. The tax2tree method used to generate draft annotations for Greengenes uses the *F*-measure to assign a candidate internal node for each named taxon. The *F*-measure is designed to balance false positives and false negatives equally as a heuristic for resolving tree conflicts. This design implicitly weights true and false positives for a taxon according to how many leaves it has, and will therefore tend to over-predict taxa with many reference sequences similarly to consensus methods.

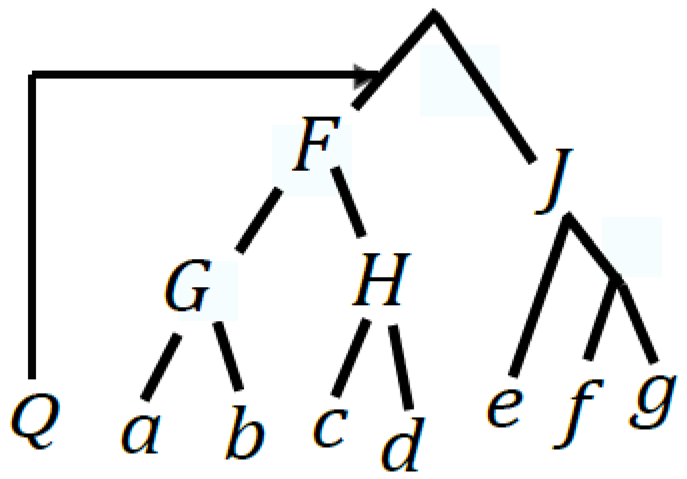

Fig. SN3.1. Example tree illustrating prediction errors by consensus methods.
