# Supplementary material for: Accuracy of taxonomy prediction for 16S rRNA and fungal ITS sequences
Source: PeerJ. 2018 Apr 18;6:e4652. doi: 10.7717/peerj.4652 (PMC5910792; doi:10.7717/peerj.4652)
Supplement: Supplemental Information 3 [file peerj-06-4652-s003.pdf]

# Accuracy of taxonomy prediction for 16S rRNA and fungal ITS sequences

Robert C. Edgar

***Supplementary Note 3: Towards realistic estimation of prediction accuracy***

The practical importance of missing data in reference databases is apparent from the divergence between OTU sequences and the databases used by taxonomy prediction algorithms. From this perspective, benchmarks are realistic if the identity distributions of test/training set pairs are similar to distributions observed in practice, and most previous benchmarks are shown by the results presented here to be unrealistic by this standard. The TAXXI implementation of cross-validation by identity makes progress towards more realistic measurement of taxonomy prediction accuracy, while also demonstrating that a single metric cannot reliably predict performance on an arbitrary query set because identity distributions vary, and prediction performance varies with identity. This can be addressed by modeling identity distributions found in environmental samples. Any given distribution, say soil vs. BLAST16S (Fig. 2 in the main text), can be approximately reproduced by constructing a test/training set pair. A benchmark could select a few representative datasets such as those in Table 1 (main text) and construct separate test/training pairs which approximate the distributions of each dataset. Metrics on these pairs would then give realistic indications of accuracy for typical well-studied and less-studied environments. A drawback of this approach is that the top-hit identity distributions (THIDs) are highly variable in practice, and the THID of OTUs obtained in an experiment might be quite different from the benchmark datasets. A more complicated but

more flexible alternative is to construct test/training pairs with all top-hit identities $d$=100, 99, 98%... to some reasonable minimum value, say 70%. The number of known and novel OTUs for any dataset can be estimated by Eqs. 4 & 5 in the main text. This enables the total number of correct predictions and mis-, under- and over-classification errors to be estimated for any dataset by summing over identities. For example, if $OCR_d(r)$ is the measured over-classification rate at rank $r$ and identity $d$, then $L^{est}_d(r)$, the estimated number of novel OTUs, can be calculated by Eq.6 in the main text and the estimated number of over-classified OTUs is:

$$N^{OC}_d(r) = L^{est}_d(r)\ OCR_d(r). \qquad \text{(Eq.SN2.1)}$$

Then, the estimated total number of over-classified OTUs at rank $r$ is obtained by summing over identities:

$$N^{OC}(r) = \Sigma_d\ N^{OC}_d(r). \qquad \text{(Eq.SN2.2)}$$

In this approach, it is computationally expensive to measure metrics for each rank and each identity (e.g., $OCR_d(r)$). However, these metric values are independent of the query set and can therefore be stored in tables which enable true positive and error rates to be easily estimated for representative datasets and for new datasets such as OTUs obtained in a sequencing experiment.
