# Supplementary material for: Accuracy of taxonomy prediction for 16S rRNA and fungal ITS sequences
Source: PeerJ. 2018 Apr 18;6:e4652. doi: 10.7717/peerj.4652 (PMC5910792; doi:10.7717/peerj.4652)
Supplement: Supplemental Information 4 — Simple example illustrating the method for calculating LCR probabilities. An all-vs-all distance matrix is constructed containing pair-wise sequence identities. Here, there are three different identities indicated by colors: green = 100%, yellow = 95% and orange = 90%. The lowest common rank (LCR) is determined for each pair by comparing taxonomy annotations. In this example, ranks are species (S), genus (G) and family (F). For each identity, the corresponding set of pairs is identified. There are three such sets, one for each of the three identities. For a given identity, the LCR frequency for a rank is calculated as the fraction of pairs having that rank. For example, there are eight pairs with 90% identity. Of these, there are no pairs with LCR = species, two pairs with LCR = genus, and six with LCR = family, so P(S|90%) = 0/8, P(G|90) = 2/8 and P(F|90) = 6/8. [file peerj-06-4652-s004.pdf]

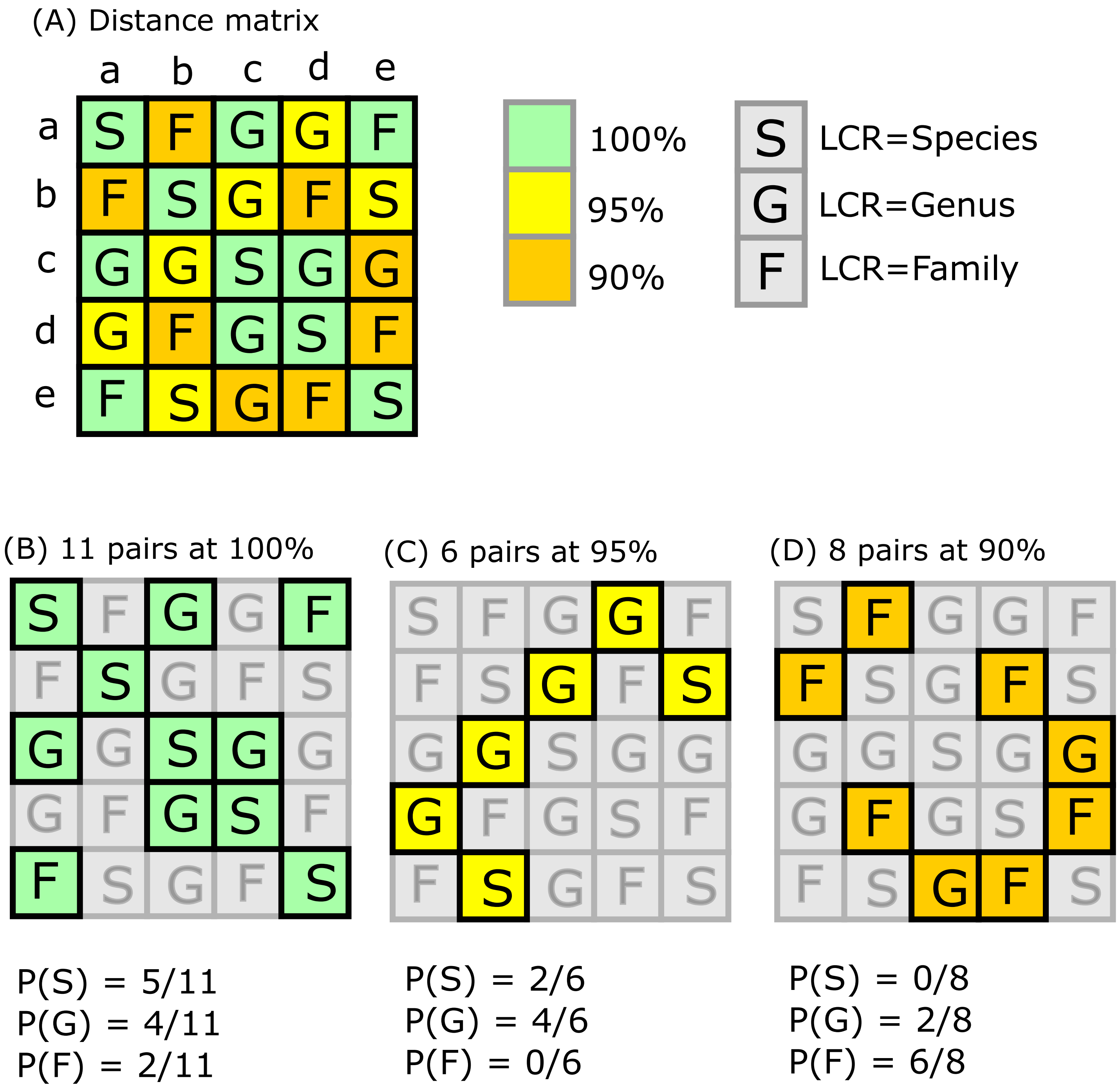

(A) Distance matrix
a b c d e
a S F G G F
b F S G F S
c G G S G G
d G F G S F
e F S G F S
100%
95%
90%
S LCR=Species
G LCR=Genus
F LCR=Family
(B) 11 pairs at 100%
(C) 6 pairs at 95%
(D) 8 pairs at 90%
P(S) = 5/11
P(G) = 4/11
P(F) = 2/11
P(S) = 2/6
P(G) = 4/6
P(F) = 0/6
P(S) = 0/8
P(G) = 2/8
P(F) = 6/8
