# Supplementary figures and images for: Accuracy of taxonomy prediction for 16S rRNA and fungal ITS sequences
Source: PeerJ. 2018 Apr 18;6:e4652. doi: 10.7717/peerj.4652 (PMC5910792; doi:10.7717/peerj.4652)

(a) Human gut vs. GG97

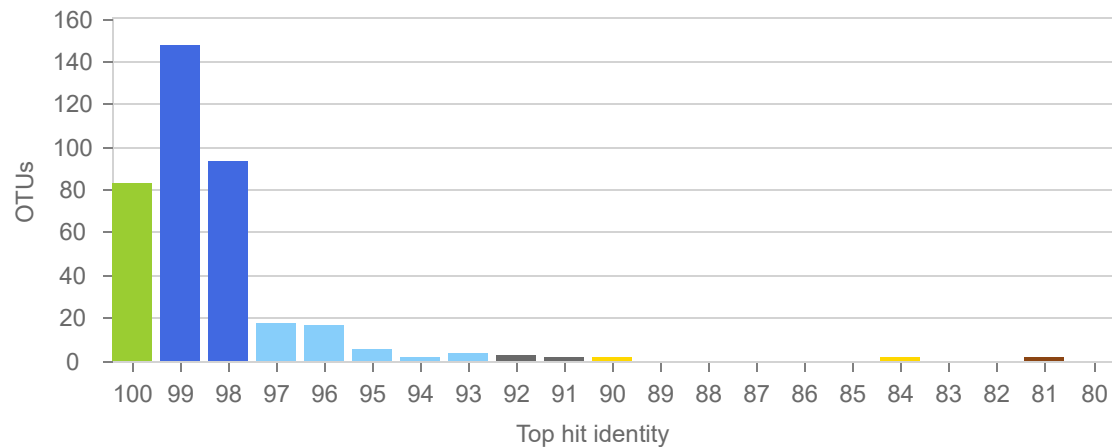

(b) Microbial mat vs. GG97

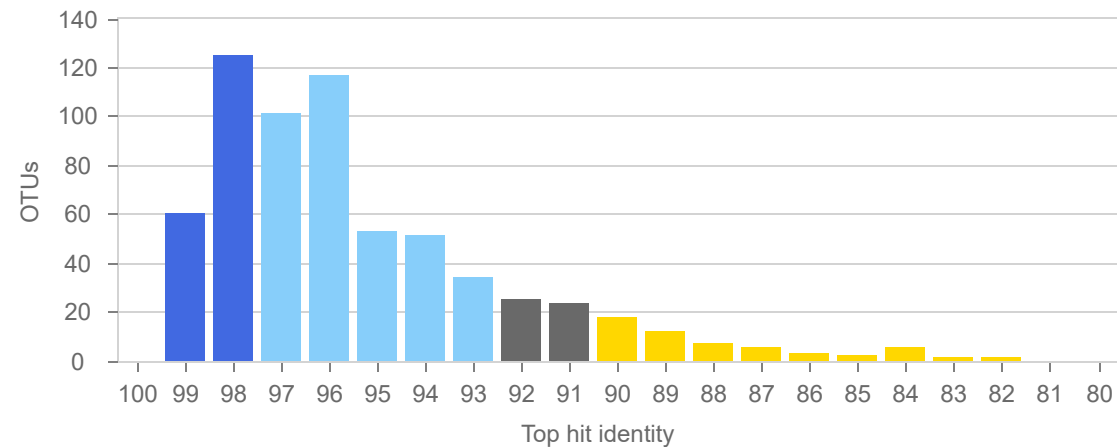

(c) Soil vs. GG97

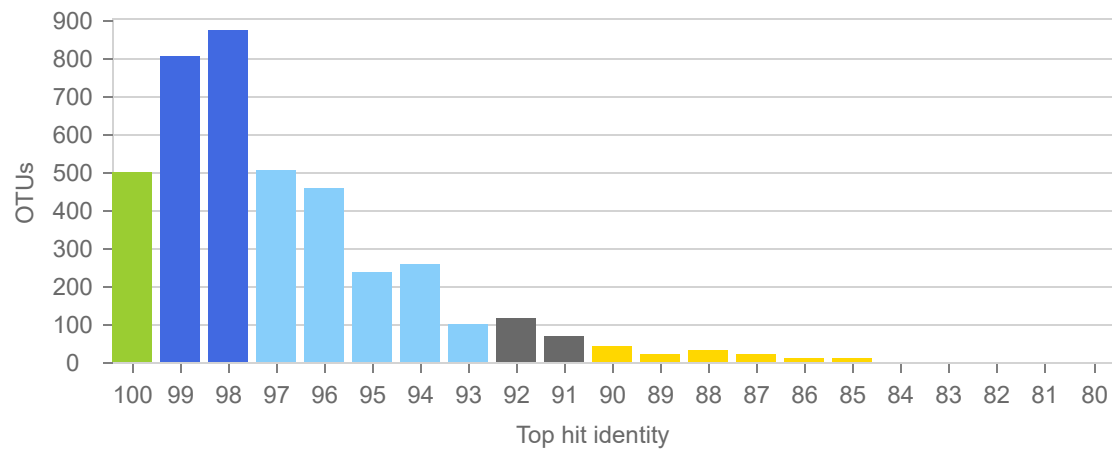

(d) Soil vs. UNITE

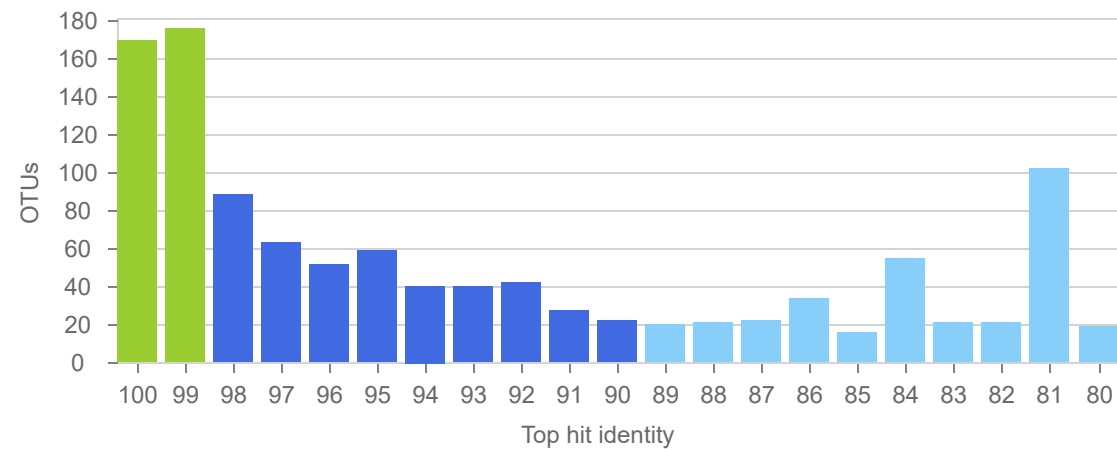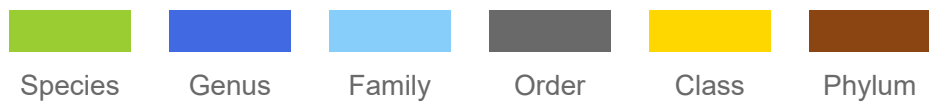

Supplement: Supplemental Information 5 — Histogram (a) is human gut vs. GG97, (b) is soil vs. GG97, (c) is microbial mat vs. GG97 and (d) is soil vs. UNITE. [file peerj-06-4652-s005.pdf]

## Bokulich F

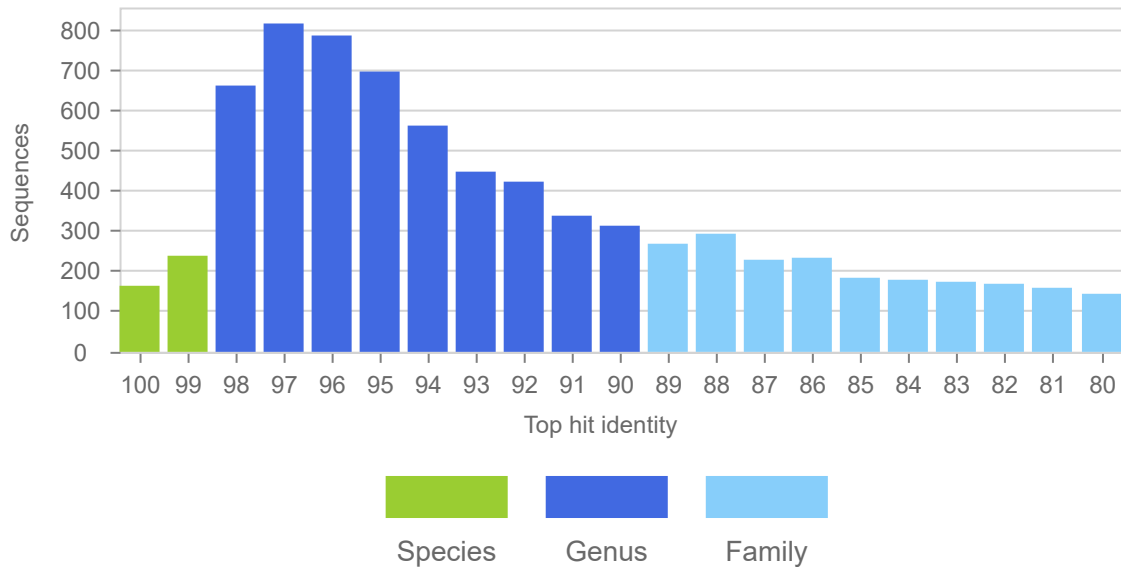

Supplement: Supplemental Information 6 — Histogram bars are colored according to the most probable lowest common rank. [file peerj-06-4652-s006.pdf]

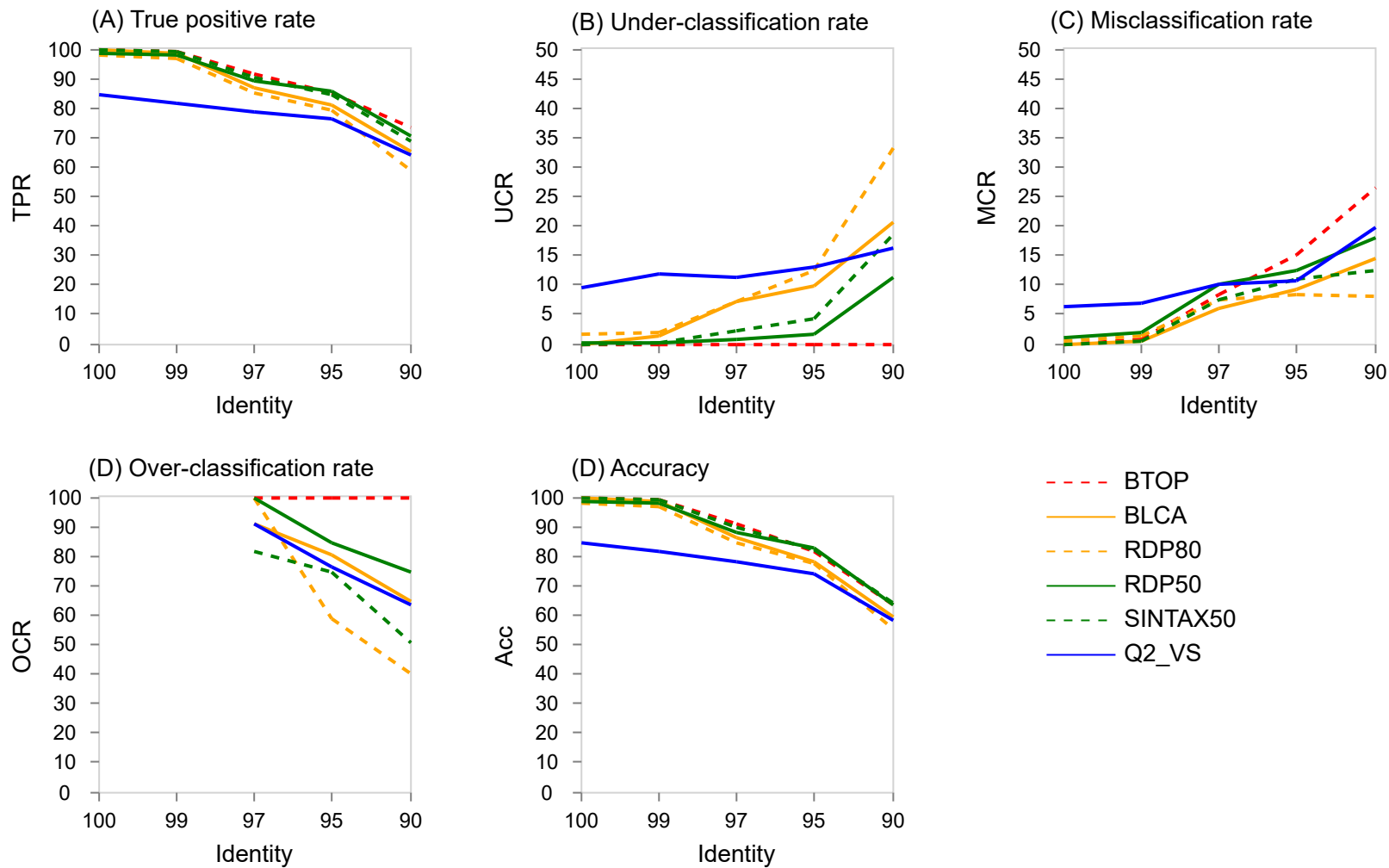

Supplement: Supplemental Information 7 — Performance metrics for genus predictions by some of the tested methods on WITS (y axis) plotted against the top-hit identity of test-training set pairs (x axis). Metrics are defined in the main text; (a) OCR (over-classification rate), (b) UCR (under-classification rate), (c) MCR (misclassification rate), (d) Acc (accuracy) and (e) TPR (true-positive rate). [file peerj-06-4652-s007.pdf]
